# Recurrent and Long-Term Oceanic Anoxia Contributed to Aborted Biotic Recovery Following the Permian–Triassic Crisis

**DOI:** 10.3390/biology15030237

**Published:** 2026-01-27

**Authors:** Wenhao Li, Bowei Yuan

**Affiliations:** 1 National Key Laboratory of Deep Oil and Gas, China University of Petroleum (East China), Qingdao 266580, China; 2 School of Geosciences, China University of Petroleum (East China), Qingdao 266580, China

**Keywords:** carbon isotopes, sulfur isotopes, pyrite, biotic recovery, oceanic anoxia, Permian–Triassic crisis, Early Triassic

## Abstract

The influence of ocean chemistry on Early Triassic biotic recovery is poorly understood in the Chaohu Area. Here, we evaluate the influence of ocean chemistry following the Permian–Triassic crisis using pyrite content, δ^13^C_org_, and S isotopic composition of pyrite. The pyrite content, V/(V + Ni) ratio, and S isotopic composition of pyrite in the Early Triassic from the northern Pingdingshan section of the Chaohu area in eastern China reveal recurrent and long-term ocean anoxia and two episodes of oxic conditions that occurred in the earliest Spathian and the late early Spathian. A positive δ^13^C_org_ shift of ~4‰ around the Smithian/Spathian boundary (SSB) in the lowermost Spathian was associated with significant biotic recovery, coincident with a positive δ^34^S excursion of ~25‰ and a low V/(V + Ni) ratio. The results suggest that the oxic conditions contributed to this recovery. Enhanced global ocean circulation during the SSB climate cooling may also have promoted this recovery. Frequent environmental perturbations may have aborted the biotic recovery, although the second episode of oxic conditions occurred in the late early Spathian. Sustained recovery did not appear in the Early Triassic because of the recurrent and long-term ocean anoxia.

## Simple Summary

The link between ocean chemistry and Early Triassic biotic recovery in the Chaohu Area remains unclear. Geochemical analyses (pyrite content, δ^13^C_org_, pyrite S isotopes, and V/(V + Ni) ratios) of the northern Pingdingshan section reveal recurrent long-term ocean anoxia in this period, interrupted by two oxic intervals in the early Spathian. A positive δ^13^C_org_ shift (~4‰) at the Smithian/Spathian boundary was associated with significant biotic recovery due to oxic conditions, coincident with a positive δ^34^S excursion (~25‰) and low V/(V + Ni) ratios. Although the second episode of oxic conditions occurred in the late early Spathian, frequent environmental perturbations may have aborted the biotic recovery. Recurrent and long-term ocean anoxia made sustained recovery impossible in the Early Triassic.

## 1. Introduction

Oceanic anoxia is considered to be the main contributor to the end-Permian mass extinction [1,2,3,4,5,6,7]. However, whether oceanic anoxia controlled the biotic recovery in the Early Triassic remains uncertain. Previous research has shown that the initial biological recovery began just after the end-Permian crisis [8,9,10,11]; however, the full marine ecosystem recovery occurred during the Middle Triassic [12,13]. Sustained euxinia or anoxia may explain the protracted recovery in the Early Triassic [6,14,15,16,17,18], although other factors like global warming caused by elevated atmospheric CO_2_ may have affected some marine invertebrates in the Early Triassic [19]. The size distribution of framboidal pyrites and S-isotope of the pyrites data from the East Greenland Basin suggest that the oceanic anoxia that developed after the end-Permian extinction event may have delayed the biotic recovery during the Early Triassic [20]. Zhou et al. [21] proposed that the incursion of euxinic waters into the surface waters delayed marine ecosystem recovery during the Early Triassic. We show that the northern Pingdingshan section of the Chaohu Area in eastern China provides strong evidence for intervals of ocean oxidation and recurrent, long-term anoxia in the Early Triassic based on the data of pyrite content, V/(V + Ni) ratio, and δ^13^C_org_ and S isotopic composition of pyrite. The recurrent and long-term oceanic anoxia played a significant role in delaying the Early Triassic recovery.

## 2. Samples and Methods

### 2.1. Sample Information

**Figure 1:**
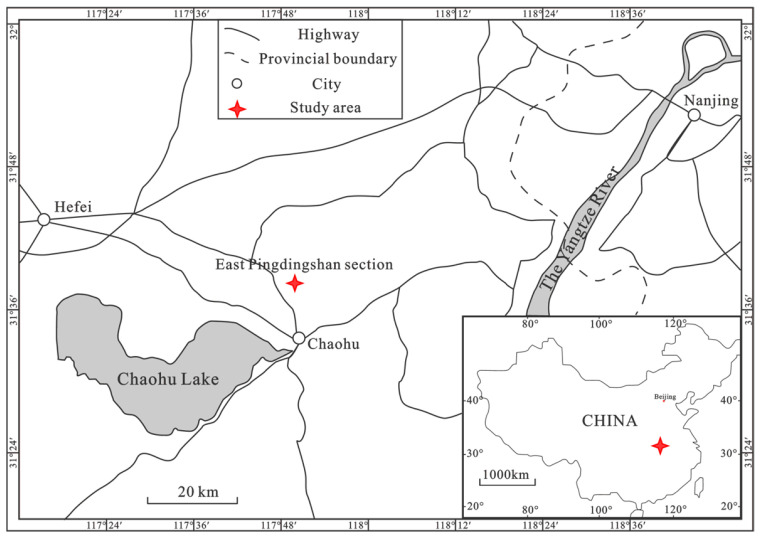
Location of northern Pingdingshan section of the Chaohu area, Anhui Province, eastern China.

**Figure 2:**
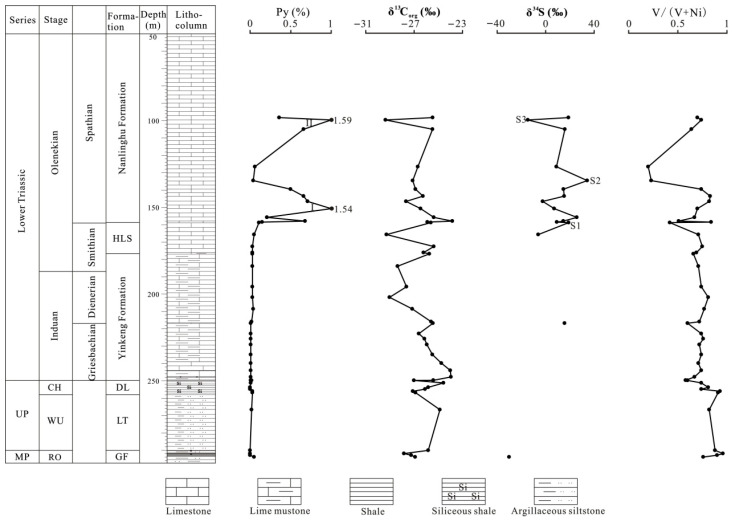
Pyrite content, V/(V + Ni), and δ^34^S and δ^13^C_org_ values for the northern Pingdingshan section of the Chaohu area, eastern China. MP = Middle Permian, UP = Upper Permian, RO = Roadian-Wordian, WU = Wuchiapingian, CH = Changhsingian, GF = Gufeng Formation, LT = Longtan Formation, DL = Dalong Formaion, and HLS = Helongshan Formation.

Forty-two samples numbered N1 to N42 from the Middle Permian Gufeng Formation to the Lower Triassic Nanlinghu Formation were collected from the northern Pingdingshan section of the Chaohu area in Anhui Province, eastern China (Figure 1, Table 1). Unabridged Upper Permian to Lower Triassic was developed in this area, which makes it a typical area for Permain–Triassic research. Mudstones and siliceous mudstones, silty mudstones, and siliceous shales (or mudstones) developed in the Middle Permian Gufeng Formation, Upper Permian Longtan, and Dalong Formation, respectively (Figure 2). Calcareous mudstones and limestones (containing laminated calcareous mudstones) primarily developed in the Lower Triassic Yinkeng and Helongshan Formations, respectively, while limestones with laminated shales developed in the Lower Triassic Nanlinghu Formation (Figure 2).

### 2.2. Methods

Samples were ground to a 200-mesh particle size using an agate mortar and pestle. Detailed experimental conditions and operational protocols for trace element analysis were previously documented in the work of Li et al. [22]. Pyrite content was quantified based on the concentration of pyrite-bound sulfur (FeS_2_), which was extracted using a CrCl_2_ reduction method and precipitated as Ag_2_S in silver nitrate trapping solutions [23]. For the determination of pyrite sulfur isotopic compositions (δ^34^S), the Ag_2_S precipitates derived from the aforementioned CrCl_2_ reduction procedure were analyzed using a Thermo Fisher Scientific Delta V Plus isotope ratio mass spectrometer (IRMS) coupled to a Flash elemental analyzer (Thermo Fisher Scientific, Waltham, MA, USA). The δ^34^S values are reported relative to the VCDT standard. Analytical precision was evaluated through replicate measurements of three international reference materials as follows: IAEA S1 (−0.3‰), IAEA S2 (+22.65‰), and IAEA S3 (−32.5‰), with the resultant precision better than ±0.2‰ (1σ).

Organic carbon isotopic compositions were assayed using a Finnigan Delta^Plus^ XL IRMS instrument connected to a CE flash^1112^ EA via a ConfloIII interface (Finnigan MAT, Bremen, Germany). Samples were subjected to high-temperature combustion under a helium carrier gas atmosphere, converting organic carbon into carbon dioxide (CO_2_). The generated CO_2_ was isolated via chromatographic separation before being introduced into the mass spectrometer for isotopic analysis. δ^13^C values are reported relative to VPDB standard. Each sample was analyzed a minimum of two times, and the variation between replicate measurements did not exceed 0.3‰. The final δ^13^C result for each sample was defined as the average value of the duplicate analyses.

## 3. Results and Discussion

### 3.1. Redox Conditions During the Permian–Triassic Crisis

Pyrite is commonly distributed in the Late Permian to Early Triassic strata of the Chaohu area [24,25]. Samples from the Gufeng, Longtan, Dalong, Yinkeng, and Helongshan Formations exhibit extremely low pyrite contents, with nearly all the samples containing <0.1% pyrite (Table 1, Figure 2), which may indicate non-euxinic conditions during the Roadian–Wordian to the Smithian. Pyrite contents for the samples in the Lower Triassic Nanlinghu Formation vary between 0.04 and 1.59%, with an average of 0.60% (Table 1, Figure 2). The pyrite contents exhibit two clear shifts, which may suggest two episodes of euxinic conditions (episodes I and II) during the Spathian (Figure 2).

V/(V + Ni) ratios are commonly used to discuss redox conditions because V becomes more enriched than Ni in anoxic environments [26]. A V/(V + Ni) ratio >0.60 may suggest anoxic bottom-water conditions [27,28]. Samples from the Gufeng, Dalong, Longtan, and Yinkeng Formations are characterized by high V/(V + Ni) ratios of >0.60 (only one <0.60%, 0.58), indicating predominantly anoxic bottom-water conditions during the Roadian–Wordian to the early Smithian. The V/(V + Ni) ratios in the Helongshan and Nanlinghu Formations range from 0.42 to 0.75 and 0.20 to 0.84, respectively, with mean values of 0.64 and 0.64 (Table 1, Figure 2), respectively, suggesting brief oxic depositional conditions during the Spathian, although most time periods exhibit anoxic bottom-water conditions. Similar findings were also presented by Du et al. [29].

Pyrite sulfur isotopes were introduced to reconstruct the ocean chemistry of the Phanerozoic biodiversity and extinction [15,20,30,31,32]. We used δ^34^S of pyrite to evaluate the influence of ocean chemistry during the Permian–Triassic crisis. Only two δ^34^Ss for pyrite values were detected from the Gufeng to the Yinkeng Formation due to the extremely low pyrite content (Figure 2). Figure 2 demonstrates that the δ^34^S values exhibit a wide range from the late Smithian to the middle Spathian. A positive δ^34^S excursion occurs from –6.0‰ to +18.9‰ (S1 in Figure 2) in the lowermost Spathian. This positive δ^34^S excursion may be associated with the short-lived oxic bottom-water conditions suggested by a lower V/(V + Ni) ratio (0.42) (Figure 2). Tian et al. also reported this oxic event during this interval [33]. The δ^34^S values first exhibit a stepwise negative excursion and then a stepwise positive excursion in the early Spathian, which reaches +34.2‰ in the late early Spathian (S2 in Figure 2). This positive excursion suggests oxic bottom-water conditions, which are also evidenced by the V/(V + Ni) ratio and pyrite content. The δ^34^S values exhibit a stepwise negative trend from the late early Spathian to the middle Spathian. A negative δ^34^S shift of ~30‰ is exhibited in the middle Spathian (S3 in Figure 2) which is associated with euxinia during episode II.

### 3.2. Biotic Recovery During the Early Triassic

**Figure 3:**
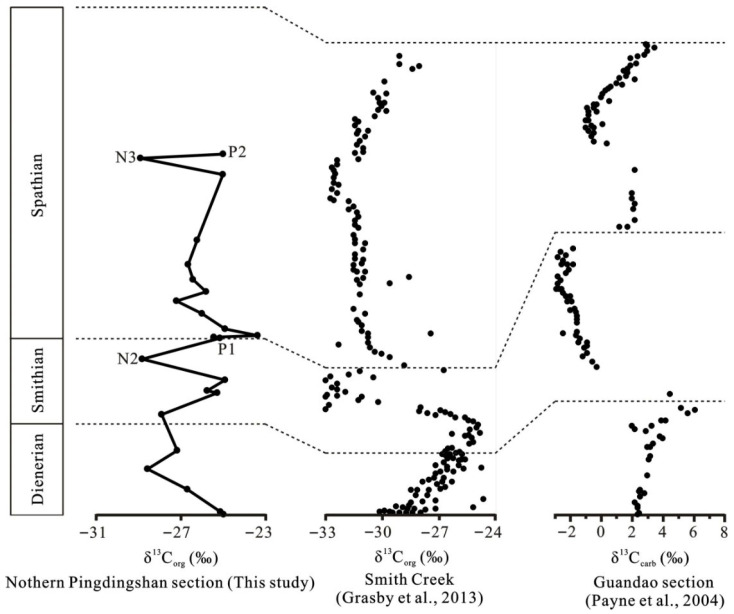
Correlations between the δ^13^C profiles of representative sections in the world during the Permian–Triassic biotic crisis [10,34].

In the northern Pingdingshan section of the Chaohu area, the δ^13^C_org_ values display stable characteristics in the Roadian–Wordian, followed by a positive excursion of ~2‰ in the Roadian–Wordian to Wuchiapingian boundary. The δ^13^C_org_ values exhibit a stepwise positive shift in the Changhsingian, followed by a 2–3‰ decline in the Permian–Triassic boundary. There was a brief stepwise positive excursion in the Early Griesbachian, followed by a continued decline in δ^13^C_org_ through the Early Griesbachian to Middle Dienerian, suggesting that the initial biotic recovery after the Permian–Triassic crisis was winding or aborted. The sustained anoxia evidenced by V/(V + Ni) ratios during Early Triassic may have been responsible for the aborted initial recovery. There is a 4‰ negative δ^13^C_org_ excursion in the late Smithian. The similarly negative excursions of δ^13^C_org_ in the late Smithian are also reported in the Smith Creek section of the Sverdrup Basin in Canada [10], Guandao section in Guizhou, China [34], and Zal section in Iran [35]. The δ^13^C_org_ excursion ranges reach up to 9‰ in the Smith Creek section (Figure 3). This negative shift is associated with anoxic conditions that resulted in the Smithian ammonoid and conodont extinction [9,36]. A high temperature was also a critical contributor to the end-Smithian crisis [37]. Climatic warming dating from the earliest Smithian and sea surface temperatures in the late Smithian may have reached a peak of ~40 °C based on the conodont oxygen isotopes [37,38].

An important recovery occurred in the early Spathian due to oxic conditions, as evidenced by the positive δ^13^C_org_ shift that occurred around the Smithian/Spathian boundary (SSB) in the lowermost Spathian, which coincided with a positive δ^34^S excursion of ~25‰ and low V/(V + Ni) ratio (0.42) (Figure 2). This positive δ^13^C_org_ shift, which we observed in the lowermost Spathian from the northern Pingdingshan section, is consistent with organic carbon isotopes from the Smith Greek section [10] and inorganic carbon isotopes from the Guandao section [34] (Figure 3). The organic carbon isotope data in the Salt Range and Surghar Range sections also recorded this positive shift [39]. Therefore, the positive excursion of δ^13^C_org_ and δ^13^C_carb_ in the lowermost Spathian was global. We believe that the biotic recovery in the earliest Spathian can be attributed to the oxic conditions observed in this study. The climatic cooling event from the latest Smithian to early Spathian that enhanced the global ocean circulation [40] and promoted marine productivity [41] also contributed to this recovery. Two episodes of euxinic conditions occurred in the early and middle Spathian in this study area. Although the second oxic event occurred in the late early Spathian, as evidenced by the positive δ^34^S excursion and low V/(V + Ni) ratio, biotic recovery did not occur because of the environmental perturbations. A ~4‰ decrease in the δ^13^C_org_ excursion occurred in the middle Spathian and coincided with a negative δ^34^S excursion of ~30‰ during episode II (Figure 2). This result suggests that ocean anoxia or euxinia contributed to the aborted biotic recovery. The Yiwagou section from the Qinling Sea records the anoxic environment in the Early Spathian [42]. A similar negative δ^13^C_org_ shift in the middle Spathian also exists in the Smith Greek and Guandao sections (Figure 3).

## 4. Conclusions

The northern Pingdingshan section of the Chaohu area, eastern China, provides strong evidence for intervals of ocean oxidation and recurrent, long-term anoxia that controlled biotic recovery in the Early Triassic. The initial biotic recovery after the Permian–Triassic crisis was winding or aborted because of the sustained ocean anoxia observed in this study area. A global biotic recovery in the earliest Spathian is attributed to oxic conditions and enhanced global ocean circulation during the SSB climate cooling. Although the oxic environment interval occurred in the late early Spathian in the northern Pingdingshan section, the negative δ^13^C_org_ excursion event indicative of anoxic conditions reveals that the environmental perturbations restrained the biotic recovery. This similar negative δ^13^C_org_ excursion event also exists in the Smith Greek (Arctic Canada) and Guandao (Guizhou Area of China) sections in the late early Spathian. Recurrent and long-term ocean anoxia made sustained recovery impossible in the Early Triassic.

## Figures and Tables

**Table 1:** The distribution of Pyrite content, V/(V + Ni), and δ^34^S and δ^13^C_org_ values for the northern Pingdingshan section of the Chaohu area in Anhui Province, eastern China.

Sample	Formation	FePy (%)	δ^13^C_org_ (‰)	δ^34^S (‰)	V/(V + Ni)
JD-1	Gufeng	0.05	−26.90	−30.1	0.76
JD-2	Gufeng	0.00	−27.23	/	0.90
JD-3	Gufeng	0.00	−27.83	/	0.96
JD-4	Gufeng	0.00	−25.83	/	0.88
JD-5	Longtan	0.02	−24.86	/	0.82
JD-6	Dalong	0.03	−26.88	/	0.91
JD-7	Dalong	0.03	−27.10	/	0.93
JD-8	Dalong	0.00	−26.09	/	0.74
JD-9	Dalong	0.00	−25.83	/	0.81
JD-10	Dalong	0.01	−24.56	/	0.74
JD-11	Dalong	0.02	−27.01	/	0.60
JD-12	Yinkeng	0.01	−25.40	/	0.58
JD-13	Yinkeng	0.01	−23.95	/	0.67
JD-14	Yinkeng	0.01	−24.00	/	0.74
JD-15	Yinkeng	0.01	−24.74	/	0.71
JD-16	Yinkeng	0.01	−25.48	/	0.74
JD-17	Yinkeng	0.01	−25.94	/	0.72
JD-18	Yinkeng	0.01	−26.14	/	0.76
JD-19	Yinkeng	0.01	−26.61	/	0.74
JD-20	Yinkeng	0.01	−25.43	15.7	0.60
JD-21	Yinkeng	0.02	−25.58	/	0.72
JD-22	Yinkeng	0.04	−27.14	/	0.77
JD-23	Yinkeng	0.03	−29.02	/	0.81
JD-24	Yinkeng	0.03	−27.63	/	0.74
JD-25	Yinkeng	0.03	−28.36	/	0.71
JD-26	Yinkeng	0.03	−25.73	/	0.66
JD-27	Helongshan	0.03	−26.21	/	0.69
JD-28	Helongshan	0.03	−25.37	/	0.75
JD-29	Helongshan	0.05	−29.28	−6.0	0.71
JD-30	Helongshan	0.11	−25.61	18.9	0.42
JD-31	Nanlinghu	0.15	−25.89	9.3	0.84
JD-32	Nanlinghu	0.68	−23.83	14.6	0.51
JD-33	Nanlinghu	0.21	−25.37	25.7	0.67
JD-34	Nanlinghu	1.54	−26.46	7.1	0.70
JD-35	Nanlinghu	0.71	−27.66	−2.4	0.82
JD-36	Nanlinghu	0.66	−26.26	15.2	0.83
JD-37	Nanlinghu	0.50	−26.88	14.7	0.74
JD-38	Nanlinghu	0.04	−27.11	34.2	0.23
JD-39	Nanlinghu	0.06	−26.68	8.9	0.20
JD-40	Nanlinghu	0.66	−25.47	15.8	0.64
JD-41	Nanlinghu	1.59	−29.36	−14.7	0.74
JD-42	Nanlinghu	0.36	−25.46	18.8	0.70

## Data Availability

The data presented in this study are available on request from the corresponding author.
